# nZVI Impacts Substrate Conversion and Microbiome Composition in Chain Elongation From D- and L-Lactate Substrates

**DOI:** 10.3389/fbioe.2021.666582

**Published:** 2021-06-15

**Authors:** Carlos A. Contreras-Dávila, Johan Esveld, Cees J. N. Buisman, David P. B. T. B. Strik

**Affiliations:** Environmental Technology, Wageningen University & Research, Wageningen, Netherlands

**Keywords:** zero-valent iron, carboxylates, n-butyrate, n-caproate, lactate racemization, lactate isomerization, lactate isomer metabolism, microbial chain elongation

## Abstract

Medium-chain carboxylates (MCC) derived from biomass biorefining are attractive biochemicals to uncouple the production of a wide array of products from the use of non-renewable sources. Biological conversion of biomass-derived lactate during secondary fermentation can be steered to produce a variety of MCC through chain elongation. We explored the effects of zero-valent iron nanoparticles (nZVI) and lactate enantiomers on substrate consumption, product formation and microbiome composition in batch lactate-based chain elongation. In abiotic tests, nZVI supported chemical hydrolysis of lactate oligomers present in concentrated lactic acid. In fermentation experiments, nZVI created favorable conditions for either chain-elongating or propionate-producing microbiomes in a dose-dependent manner. Improved lactate conversion rates and n-caproate production were promoted at 0.5–2 g nZVI⋅L^–1^ while propionate formation became relevant at ≥ 3.5 g nZVI⋅L^–1^. Even-chain carboxylates (n-butyrate) were produced when using enantiopure and racemic lactate with lactate conversion rates increased in nZVI presence (1 g⋅L^–1^). Consumption of hydrogen and carbon dioxide was observed late in the incubations and correlated with acetate formation or substrate conversion to elongated products in the presence of nZVI. Lactate racemization was observed during chain elongation while isomerization to D-lactate was detected during propionate formation. *Clostridium luticellarii*, *Caproiciproducens*, and *Ruminococcaceae* related species were associated with n-valerate and n-caproate production while propionate was likely produced through the acrylate pathway by *Clostridium novyi*. The enrichment of different potential n-butyrate producers (*Clostridium tyrobutyricum*, *Lachnospiraceae*, *Oscillibacter*, *Sedimentibacter*) was affected by nZVI presence and concentrations. Possible theories and mechanisms underlying the effects of nZVI on substrate conversion and microbiome composition are discussed. An outlook is provided to integrate (bio)electrochemical systems to recycle (n)ZVI and provide an alternative reducing power agent as durable control method.

## Introduction

Production of biochemicals from renewables is of outmost importance to reduce anthropogenic impact on the environment. Carboxylates are platform chemicals that are produced by chemical or biological means. Short-chain carboxylates (SCC, up to 5 carbons) and methane are commonly observed in biological conversion of organics during anaerobic fermentation whereas medium-chain carboxylates (MCC, 6–12 carbons) are produced by specialized chain-elongating bacteria in the presence of reduced compounds in the so-called chain elongation process (Angenent et al., 2016). The metabolic energy to drive the chain elongation process is supplied by a variety of electron donors (e.g., alcohols, lactate, sugars) that can be obtained from waste biomass materials (Zhu et al., 2015; Angenent et al., 2016; Contreras-Dávila et al., 2020). Lactate is produced as one or a mixture of the two enantiomeric forms (D-lactate or L-lactate) depending on the culture conditions (Hofvendahl and Hahn-Hägerdal, 2000). Numerous (bio)process reactions can occur within lactate-based chain elongation microbiomes (Table 1). Lactate may be interconverted between the two enantiomeric forms by lactate racemase (Lar) (Eq. 1). Under anaerobic conditions, lactate is first oxidized to pyruvate by confurcating lactate dehydrogenase which transfer electrons from lactate and reduced ferredoxin (Fd_*red*_) to reduce NAD (Buckel and Thauer, 2018). Then, pyruvate is further converted to acetyl-CoA and carbon dioxide (CO_2_) through pyruvate:ferredoxin oxidoreductase (PFOR) (Liu et al., 2020). This two-step lactate oxidation to acetate (Eq. 2) yields electrons and carbon for the reverse-β-oxidation (RBO) pathway (Liu et al., 2020). In the RBO pathway, acetate is elongated with two carbons from acetyl-CoA to even-chain carboxylates such as n-butyrate (nC4) and n-caproate (nC6). Additionally, odd-chain carboxylates such as n-valerate (nC5) may be produced from propionate elongation (Eqs. 4–8). Propionate can be produced from lactate (Eq. 9) by organisms such as *Megasphaera elsdenii* (Hino and Kuroda, 1993) or propionic acid bacteria (PAB) (Seeliger et al., 2002; Gonzalez-Garcia et al., 2017).

**TABLE 1:** Thermodynamics of (bio)process reactions potentially involved in lactate-based chain elongation microbiomes and nZVI conversion.

No.	Equation	e^–^ mol transferred	ΔG^*o*^′	ΔG′	
				
			[kJ⋅reaction^–^^1^]	[kJ⋅reaction^–^^1^]	[kJ⋅e^–^ mol^–^^1^]
	**Lactate (inter)conversion**				
1	L-lactic acid ↔ D-lactic acid	–	−0.4	−0.4	–
2	lactate^–^ + H_2_O → acetate^–^ + 2 H_2_ + CO_2_	4	−9.5	−9.5	−2.38
3	lactate^–^ + Fe^2+^ + H_2_O → acetate^–^ + 2 Fe^0^ + CO_2_ + 4H^+^	4	−169.2	−135.0	−33.75
	**Lactate-based chain elongation**				
4	1.4 lactate^–^ + 0.6 acetate^–^ + H^+^ → n-butyrate^–^ + 0.8 H_2_ + 1.4 CO_2_ + 0.6 H_2_O^a^	5.6	−61.7	−70.2	−12.54
5	lactate^–^ + acetate^–^ + H^+^ → n-butyrate^–^ + CO_2_ + H_2_O^b^	4	−57.9	−66.4	−16.61
6	lactate^–^ + propionate^–^ + H^+^ → n-valerate^–^ + CO_2_ + H_2_O^b^	4	−57.8	−66.3	−16.58
7	lactate^–^ + n-butyrate^–^ + H^+^ → n-caproate^–^ + CO_2_ + H_2_O^b^	4	−57.9	−66.4	−16.61
8	2 lactate^–^ + acetate^–^ + 2 H^+^ → n-caproate^–^ + 2 CO_2_ + 2 H_2_O^c^	8	−115.7	−132.8	−16.61
	**Propionate formation**				
9	3 lactate^–^ → 2 propionate^–^ + acetate^–^ + CO_2_ + H_2_O^d^	12	−172.0	−172.0	−14.33
	**Chemical reactions involving nZVI**				
10	Fe^0^ + 2 H^+^→ Fe^2+^ + H_2_	2	−10.6	−27.7	−13.87
11	Fe^0^ + 2 H_2_O → Fe^2+^ + 2 OH^–^ + H_2_	2	−10.6	−27.7	−13.87
12	Lactyl lactate^–^ + OH^–^ → 2 lactate^–e^	–	−112.1	−103.6	−
	**Homoacetogenesis with nZVI or hydrogen**				
13	4 Fe^0^ + 2 CO_2_ + 7 H^+^ → 4 Fe^2+^ + acetate^–^ + 2 H_2_O	8	−137.6	−197.5	−24.68
14	4 H_2_ + 2 CO_2_ → acetate^–^ + 2 H_2_O + H^+^	8	−95.7	−87.2	−10.90
	**Chain elongation with nZVI or hydrogen**				
15	2 Fe^0^ + 2 acetate^–^ + 5 H^+^ → 2 Fe^2+^ + n-butyrate^–^ + 2 H_2_O	4	−69.7	−112.5	−28.13
16	2 Fe^0^ + acetate^–^ + n-butyrate^–^ + 5 H^+^ → 2 Fe^2+^ + n-caproate^–^ + 2 H_2_O	4	−69.7	−112.5	−28.13
17	2 H_2_ + 2 acetate^–^ + H^+^ → n-butyrate^–^ + 2 H_2_O	4	−48.4	−56.9	−14.23
18	2 H_2_ + acetate^–^ + n-butyrate^–^ + H^+^ → n-caproate^–^ + 2 H_2_O	4	−48.4	−56.9	−14.23

*Free energies of formation used for calculations are shown in Supplementary Table 1. All free energies of reaction are calculated considering reactants and products concentrations of 1 M or 1 atm (H_2_, CO_2_). ΔG^o^′ values are corrected for pH 7; ΔG′ for pH 5.5 according to ΔG′ = ΔG^o^ + RT⋅Y_H__+_⋅ln[H^+^] where Y_H__+_ is the reaction proton yield/consumption. Y_H__+_ = -Y_OH__–_ in Eqs. 11 and 12. ^a^Based on results from this study and similar to C. acetobutylicum (Diez-Gonzalez et al., 1995). ^b^Proposed for a reactor microbiome dominated by Ruminococcaceae bacterium CPB6 (Zhu et al., 2015). ^c^Equations 5 + 7. ^d^(Seeliger et al., 2002). ^e^(Ivanova et al., 2000).*

Several operational conditions such as pH, electron donor-to-acceptor ratio and hydrogen partial pressure can be used to steer chain elongation microbiomes (Angenent et al., 2016). The use of additional electron donors that do not act as carbon sources (e.g., hydrogen, cathodes, transition metals) can result in lower oxidation-reduction potential (ORP) and unbalanced fermentation in e.g., carboxylates and alcohol producing processes (Moscoviz et al., 2016; Wang et al., 2020). In this study, nano zero-valent iron (nZVI) was tested for steering product formation in chain elongation. Anaerobic corrosion of zero-valent iron (Eqs. 10 and 11) may decrease ORP by increasing pH or reducing anions such as NO_3_^–^ and SO_4_^2–^ present in the medium (Tratnyek et al., 2003). Together with Fe^2+^ released, reduced ORP stimulate the interconversion between the two lactate enantiomers by lactate racemase (Katagiri et al., 1961). L-lactate isomerization to D-lactate has been induced with zero-valent iron addition during lactate production in open-culture organic waste fermentation (Li et al., 2017). Lactate enantiomers interconversion could in turn impact lactate-based chain elongation product spectrum since organisms like *M. elsdenii* have been suggested to produce even-chain carboxylates from D-lactate and odd-chain carboxylates from L-lactate (Hino and Kuroda, 1993). nZVI may also donate electrons to bacteria through direct or H_2_-mediated electron transfer (Tang et al., 2019; Gong et al., 2020), potentially influencing metabolic processes such as lactate oxidation to pyruvate (Fd_*red*_-dependent lactate dehydrogenase; Weghoff et al., 2015), pyruvate decarboxylation (PFOR) (Meng et al., 2013), hydrogen formation and energy conservation (Angenent et al., 2016). These effects would in turn affect conversion rates and elongation of carboxylates. The electron donor hydrogen, for instance, improves MCC formation from lactate and food waste (Nzeteu et al., 2018; Wu et al., 2019).

Pyruvate decarboxylation by PFOR is a carbon-diverging step in lactate-based chain elongation as it results in carbon lost as CO_2_. Although lactate-elongating bacteria do not seem to metabolize CO_2_ to carboxylates (Tao et al., 2017), this could be done by other organisms in anaerobic microbiomes to increase carbon recovery. Homoacetogenic bacteria may use electrons or H_2_ to reduce CO_2_ to acetate (Eqs. 13 and 14). Acetate can then be used by chain-elongating bacteria to produce MCC. However, CO_2_ recapture would be limited to 33–50% since homoacetogenic bacteria requires four H_2_ molecules to reduce two CO_2_ to acetate (H_2_/CO_2_ ratio = 2) while the stoichiometric H_2_/CO_2_ ratio produced from lactate is 1 or 2/3 when n-butyrate or n-caproate are produced, respectively (Contreras-Dávila et al., 2020). To achieve higher carbon recoveries, additional electron donors such as hydrogen (Wu et al., 2019) or nZVI (Eq. 11) could be used. nZVI addition is reported to promote homoacetogenic activity in anaerobic digestion microbiomes (Meng et al., 2013). Lastly, nZVI may also increase substrate conversion when lactate (poly)esters are present e.g., poly-lactic acid (PLA) bioplastics or concentrated lactate solutions (Vu et al., 2005). Thus, appropriate nZVI doses should be added to adequately steer the microbiome. Recent reports on ethanol-elongating microbiomes showed improved substrate conversion to MCC with nZVI supplementation (Fu et al., 2020; Wang et al., 2020). However, nZVI effects on lactate-based chain elongation have not been studied. The aim of this work was to evaluate the effect of nZVI addition on batch lactate-based chain elongation. We hypothesized that nZVI could promote (1) lactate conversion to MCC by donating extra electrons; (2) lactate isomerization to produce even/odd carbon chains; and (3) carbon dioxide capture through acetate formation (Table 1). Abiotic tests with nZVI were done to evaluate chemical hydrolysis of lactate oligomers present in concentrated lactic acid (experiment I). Chain elongation incubations were carried out at different nZVI (experiment II) and H_2_ (experiment III) concentrations. Finally, chain elongation of enantiopure lactate (D-lactate and L-lactate separately) or a racemic mixture of both enantiomers was studied with and without nZVI (experiment IV).

## Materials and Methods

### Mineral Medium and Inoculum

Salts and vitamins were added to the experiments as in Roghair et al. (2016). The trace elements solution contained: nitrilotriacetic acid, 2.0 g⋅L^–1^; MnSO_4_⋅H_2_O, 1.0 g⋅L^–1^; Fe(SO_4_)_2_ (NH_4_)_2_⋅6H_2_O, 0.8 g⋅L^–1^; CoCl_2_⋅6H_2_O 0.2 g⋅L^–1^; ZnSO_4_⋅7H_2_O 0.2 mg⋅L^–1^; CuCl_2_⋅2H_2_O 20.0 mg⋅L^–1^; NiCl_2_⋅6H_2_O 20.0 mg⋅L^–1^; Na_2_MoO_4_⋅2H_2_O 20.0 mg⋅L^–1^; Na_2_SeO_4_, 20.0 mg⋅L^–1^; Na_2_WO_4_, 20.0 mg⋅L^–1^, and was added at 1 mL⋅L^–1^_*m*__*edium*_ according to Zhu et al. (2015). Yeast extract was added at 1 g⋅L^–1^. Biomass from a lab-scale chain elongation bioreactor converting lactate and acetate to n-caproate was used as inoculum (Contreras-Dávila et al., 2020). The lab-scale reactor contained granular activated carbon as carrier material. Inoculum samples were taken during (experiments II–III) and after (experiment IV) continuous operation (section “Experimental Set-Up”). Inoculum was added at 5% v/v after centrifugation at 10,000 rpm for 10 min and resuspension of the pellet in N_2_-bubbled demi water.

### Substrates and nZVI Material

Lactate used as electron donor in the experiments were: concentrated lactic acid (≥90%) (VWR, the Netherlands), sodium-D-lactate ≥99.0% (Sigma-Aldrich, the Netherlands) and sodium-L-lactate ≥99.0% (Sigma-Aldrich, the Netherlands). The concentrated lactic acid was a mixture of lactate monomers and oligomers (Vu et al., 2005) containing mainly L-lactic acid (≥97%, as reported by VWR); D-lactic acid was not detected in these chemicals by our lab analysis. Acetic acid 99–100% (Merck KGaA, Germany) was added as electron acceptor. The nZVI particles (Nanofer Star, Nano Iron, Czech Republic) used in this study had an average particle size of 60 ± 1.3 nm and contained 74% Fe^0^, along with 8% FeO and 18% Fe_3_O_4_ with an oxides layer thickness of 4.3 ± 0.53 nm (as reported by Nano Iron). Before application, 1 g of nZVI powder was suspended in 4 mL of N_2_-bubbled demi water. The suspension was mixed for 10 min using a high shear mixer (Ultra-Turrax T25, IKA, Germany). After this, the bottle containing the slurry was loosely closed to avoid overpressure due to hydrogen gas release and left to rest at room temperature for ∼48 h before adding it to the experiments. This procedure followed the instructions of the supplier and served to erode the protective oxides layer present on the nZVI particles.

### Experimental Set-Up

Incubations were carried out in 125 mL serum bottles using a liquid volume of 40 mL. An overview of the initial conditions in the different experiments can be found in Table 2. First, chemical hydrolysis of lactate oligomers by nZVI particles was evaluated under abiotic conditions in experiment I. Since concentrated lactic acid solutions contain lactate oligomers (Vu et al., 2005), nZVI was added at 1 and 5 g⋅L^–1^ to a 27 g⋅L^–1^ total lactate (monomers + oligomers) solution prepared with concentrated lactic acid. Hydrolysis was followed by measuring the increase in monomeric lactate concentrations. A control experiment with no nZVI was included. Experiments were done in triplicate at an initial pH of 5.5. Vitamins, yeast extract, (NH_4_)H_2_PO_4_ and trace elements were left out to prevent microbial growth.

**TABLE 2:** Overview of experimental design.

Experiment	Substrate	Lactate monomers concentration [g⋅L^–^^1^]	Lactate oligomers concentration^a^ [g⋅L^–^^1^]	Electron acceptor concentration [g⋅L^1^]	nZVI concentration [g⋅L^–^^1^]	Hydrogen [atm]
I) Chemical hydrolysis	Concentrated lactic acid	19.3	10.7	0	0 (Control)	0
	Concentrated lactic acid	19.3	10.7	0	1	0
	Concentrated lactic acid	19.3	10.7	0	5	0
II) nZVI concentrations	Concentrated lactic acid	19.3	10.7	6	0 (Control)	0
	Concentrated lactic acid	19.3	10.7	6	0.5	0
	Concentrated lactic acid	19.3	10.7	6	1	0
	Concentrated lactic acid	19.3	10.7	6	2	0
	Concentrated lactic acid	19.3	10.7	6	3.5	0
	Concentrated lactic acid	19.3	10.7	6	5	0
III) Hydrogen effect	Concentrated lactic acid	17.2	9.5	6	0	0 (Control)
	Concentrated lactic acid	17.2	9.5	6	0	0.45
	Concentrated lactic acid	17.2	9.5	6	0	1.2
	acetate + n-butyrate	0	0	6 + 9	0	0.75
IV) Lactate enantiomers	Sodium D-lactate	18	0	5	0	0
	Sodium D-lactate	18	0	5	1	0
	Sodium L-lactate	18	0	5	0	0
	Sodium L-lactate	18	0	5	1	0
	Racemic sodium lactate	18	0	5	0	0
	Racemic sodium lactate	18	0	5	1	0

*^a^Difference between total lactate added and measured lactate monomers.*

In experiment II, the effect of different nZVI concentrations (0.5, 1, 2, 3.5, and 5 g⋅L^–1^) on lactate-based chain elongation was tested in duplicate batch incubations using 30 g⋅L^–1^ total lactate and 6 g⋅L^–1^ acetic acid. This resulted in monomeric L-lactate concentrations of 19.3 ± 0.6 g⋅L^–1^ (64% of total lactate added) as expected from 90% concentrated lactic acid solutions (Vu et al., 2005). The control experiment was carried out without nZVI. The experiment with 3.5 g nZVI⋅L^–1^ was done using a 30 mL liquid volume. Since hydrogen release from nZVI corrosion could be an additional electron donor, the effect of H_2_ on lactate-based chain elongation was studied in experiment III by adding H_2_ at initial partial pressures (P_*H*__2_) of 0.45 and 1.2 atm. Acetate (5.7 g⋅L^–1^) and n-butyrate (9.3 g⋅L^–1^) elongation with H_2_ as sole electron donor (0.75 atm) was also tested in the same experiment. Lastly, to test the effect of the lactate enantiomer fed and the presence of nZVI on lactate conversion and product spectrum, fermentation of D-lactate and L-lactate with and without nZVI (1 g⋅L^–1^) was studied in triplicates in experiment IV. Enantiopure or a racemic mixture (1:1) of D- and L-lactate sodium salts were added to reach a lactate concentration of 18 g⋅L^–1^. This concentration is comparable to the monomeric lactate concentrations measured in the experiments supplied with concentrated lactic acid. Acetate was added as electron acceptor at 5 g⋅L^–1^. All the experiments were incubated in a shaker at 120 rpm and 30°C. Initial pH was adjusted to 5.5 by adding 4 M KOH and/or 1 M HCl. Headspace gas was exchanged by vacuum/filling cycles (5 times) with N_2_ in experiment I and N_2_:CO_2_ (80:20) in experiments II-IV to a final overpressure of 0.5 atm. N_2_ was partly replaced with H_2_ to reach designated P_*H*__2_ for experiment III. When pressure raised above 2.5 atm during experiments II and III, gas was manually released to bring headspace pressure down to 1.2–1.5 atm and the released gas volume was measured. Gas bags were used in experiment IV to avoid overpressure. Process performance in the incubations was evaluated based on substrate conversion (total lactate + acetate); lactate enantiomeric distribution; and electron and carbon selectivities (see Supplementary Material).

### Analytical Methods

Liquid samples were taken regularly to measure pH and soluble metabolites. Samples were centrifuged (15,000 rpm, 10 min) and stored at −20°C before metabolites analyses. Lactate (both D- and L-lactate monomers together), succinate and formate were measured by HPLC (Contreras-Dávila et al., 2020). Lactate enantiomers (D-lactate and L-lactate) were separated and quantified *via* isocratic HPLC using a chiral column. Fatty acids, alcohols and gas headspace composition (O_2_, N_2_, CO_2_, CH_4_, H_2_) were quantified by gas chromatography. For methods description see Supplementary Material. The gas production/consumption and partial pressures were estimated using headspace composition, manually measured pressure and gas released during pressure adjustments. Raw experimental data are available in the 4TU.ResearchData repository^1^.

### Microbiome Analysis

Samples were centrifuged at 10,000 rpm for 10 min and stored at −20°C for DNA extraction and sequencing. DNA was extracted from the pellets (PowerSoil DNA isolation kit) and used as template for amplifying the V3–V4 region of 16S rRNA via Illumina sequencing using the primer sets described by Klindworth et al. (2013) for simultaneous amplification of bacterial and archaean 16S rRNA. The sequences were deposited in the European Nucleotide Archive^2^ under accession number PRJEB41368. DNA sequences were processed as described previously (Contreras-Dávila et al., 2020): the DADA2 pipeline (Callahan et al., 2016) was used and the identified ASVs were submitted to the SILVA database for taxonomy assignment. Forward and reverse reads were trimmed at cycles 240 and 220, respectively, based on the quality profiles obtained. Species assignment is based on exact sequence matching. Selected sequences with non-exact match were submitted to NCBI BLAST query (megablast 16S rRNA bacterial and archaean sequences) and the percentage of identity is reported. ASVs with ≥0.05% of total counts were used for analyses. Distance-based redundancy analysis (dbRDA) was done using Bray-Curtis dissimilarity with the capscale function from the vegan package (Oksanen et al., 2019) and visualized with ggord and ggplot2 (Wickham, 2008; Beck and Mikryukov, 2020). Correlations between nZVI addition, chemical parameters and relative abundance of microbial taxa were investigated using Spearman’s rank correlation coefficient with the PerformanceAnalytics package (cor.test function-based) (Peterson et al., 2020).

## Results

### Experiment I—Chemical Hydrolysis of Lactate Oligomers With nZVI

**FIGURE 1:**
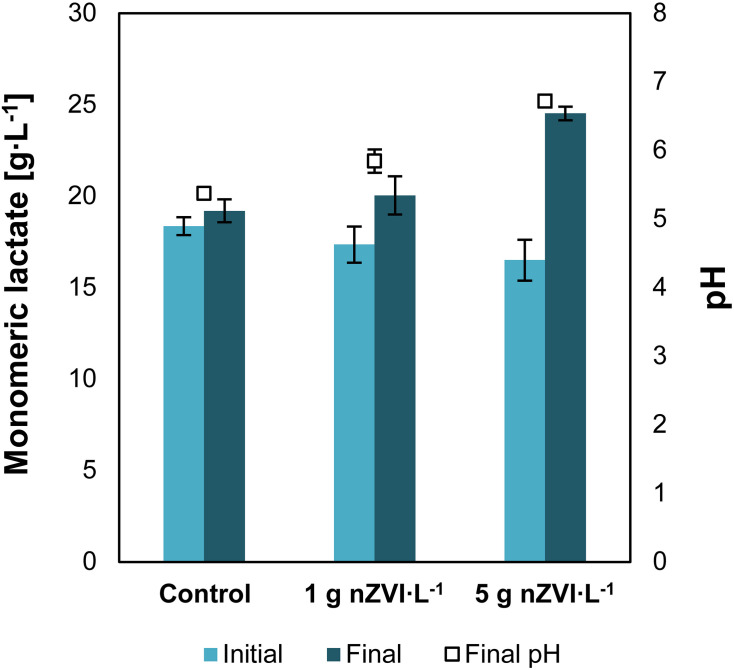
Experiment I—Increase in monomeric lactate concentrations and pH due to nZVI addition. Data shows the initial and final results after 18 days of reaction. Error bars depict ± one standard deviation.

Addition of nZVI to a solution of concentrated lactic acid and nitrogen-free medium resulted in an increase of lactic acid monomers concentration. Lactate concentrations increased from 17.4 ± 0.9 to 20.0 ± 1.0 g⋅L^–1^ when 1 g⋅L^–1^ of nZVI was added and from 16.5 ± 1.0 to 24.5 ± 0.3 g⋅L^–1^ with 5 g nZVI⋅L^–1^, showing that nZVI is capable to support hydrolysis of lactate oligomers present in concentrated lactic acid solutions (Figure 1). Lactic acid oligomerization is known to occur as acid concentrations increase. The hydroxyl and carboxyl groups in lactic acid interact with other lactate molecules resulting in intermolecular self-esterification forming lactyl lactic acid (dimer), lactyl-lactylactic acid (trimer) and so on (Vu et al., 2005). Here, addition of nZVI resulted in chemical hydrolysis of lactate oligomers as evidenced by an increase in lactate monomers concentration. The final pH at 1 and 5 g⋅L^–1^ of nZVI⋅L^–1^ was 5.8 ± 0.2 and 6.7 ± 0.1, respectively, compared to 5.4 in the control experiment. We estimated that 29 ± 19 and 88 ± 22% of lactate oligomers were hydrolyzed at 1 and 5 g nZVI⋅L^–1^, respectively. These value was 8 ± 1% for the control without nZVI.

### Experiment II and III—nZVI Addition to a Mixture of Lactate Monomers and Oligomers Steers to Microbial n-Caproate Formation

**FIGURE 2:**
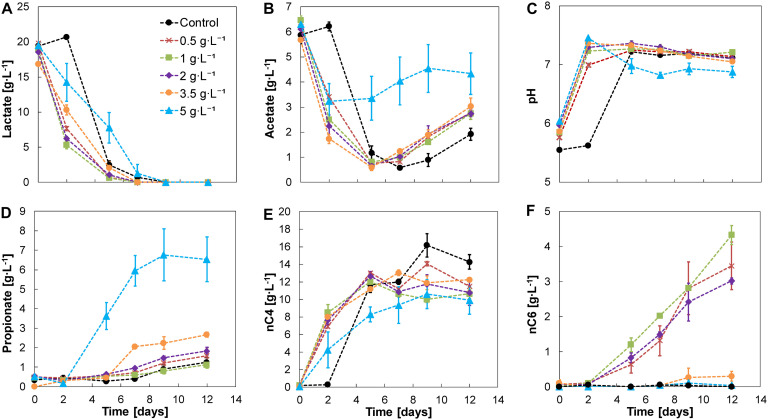
Experiment II—Substrates **(A,B)**, pH **(C)**, and metabolites **(D–F)** profile of lactate-based chain elongation with different nZVI concentrations. Error bars show duplicates absolute deviation from the average.

Adding nZVI at concentrations ranging from 0.5 to 5 g⋅L^–1^ showed no evident inhibition on chain elongation activity. Lactate was completely consumed within 5–9 days in all cases. A lag phase of 2 days was observed in the control without nZVI which was shortened in the presence of nZVI. After adaptation, a lactate-based chain elongation phase was observed where lactate and acetate were converted to mainly n-butyrate accompanied by an increase in pH and hydrogen release (days 0–7) (Figure 2 and Supplementary Figure 1). The highest lactate conversion rate was detected at 1 g nZVI⋅L^–1^ (6.87 ± 0.53 g⋅L^–1^⋅d^–1^; days 0–2), slightly higher than at 0.5 and 2 g nZVI⋅L^–1^ (6.08 ± 0.05 and 6.17 ± 0.21 g⋅L^–1^⋅d^–1^, respectively, days 0–2). Conversion rates were much lower at higher nZVI doses of 3.5 and 5 g⋅L^–1^ (3.28 ± 0.41 and 2.62 ± 1.61 g⋅L^–1^⋅d^–1^, respectively, days 0–2).

n-butyrate concentrations reached 14 ± 0.8 g⋅L^–1^ in the control experiment and were consistently lower in the presence of nZVI. With nZVI concentrations between 0.5 and 2 g⋅L^–1^, n-caproate production was promoted, reaching n-caproate concentrations of 4.3 ± 0.3 g⋅L^–1^ at 1 g nZVI⋅L^–1^ (Figure 2F). Despite apparent lactate depletion, n-caproate formation did not reach a plateau (Figure 2F) with a similar trend for n-valerate (Supplementary Figure 2). This suggests that lactate oligomers or other electron donors were used for chain elongation. A similar behavior in hydrogen partial pressure (P_*H*__2_) was observed in all tested conditions for experiment II giving no obvious relation between P_*H*__2_ and n-caproate formation (Supplementary Figure 1A). Substituting nZVI with hydrogen at 0.45 atm (amount expected from dissolution of 1 g nZVI⋅L^–1^) or 1.2 atm in experiment III did not result in n-caproate formation. Additionally, neither acetate nor n-butyrate were elongated with hydrogen as sole electron donor (Supplementary Figure 3). Further chain elongation to n-caproate was apparently limited by lactate availability. L-lactate was racemized to near-equilibrium concentrations of both D-lactate and L-lactate with 0–2 g nZVI⋅L^–1^ (Supplementary Figure 4). At 5 g nZVI⋅L^–1^ propionate formation instead of n-caproate was favored. n-butyrate formation was primarily observed in the first 2 days. pH increased to a value of 7.5 (Figure 2C) and propionate started being produced from the leftover lactate to reach 6.5 ± 1.2 g⋅L^–1^ of propionate with no clear acetate consumption thereafter. Moreover, D-lactate reached an enantiomeric excess of 71 ± 21% when substantial propionate formation was observed (Supplementary Figure 4) suggesting that propionate was formed through the acrylate pathway in which D-lactate is selectively reduced (Akedo et al., 1983; Schweiger and Buckel, 1984; Kuchta and Abeles, 1985). Fermentation with 3.5 g nZVI⋅L^–1^ exhibited a transitional behavior between chain elongation and propionate production, with similar n-valerate but less n-caproate formation compared to incubations with lower nZVI doses (Supplementary Figure 2 and Figure 2). In all conditions tested, a hydrogen consumption phase (days 7–12) was observed (Supplementary Figure 1). During this phase different processes such as chain elongation, acetate and propionate formation continued to occur which may have contributed to hydrogen consumption. H_2_ conversion through homoacetogenesis was estimated to contribute in minor proportions to acetate formation as the expected acetate production from the H_2_ consumed was lower than the measured increase in acetate concentrations (Supplementary Figure 5). However, homoacetogenesis could have been underestimated due to continuous H_2_ release from nZVI, acetate uptake for chain elongation and/or direct electron transfer during both chain elongation and hydrogen consumption phases.

**FIGURE 3:**
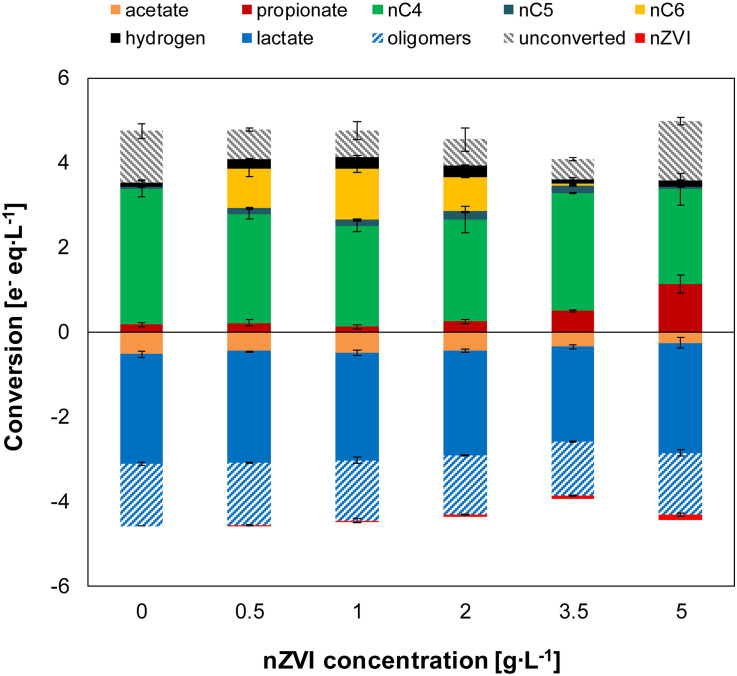
Experiment II—Net conversion by the end of the experiments at different nZVI concentrations. Oligomers were estimated as a fraction (0.36) of total lactate. The unconverted fraction shows missing electrons (from nZVI and oligomers) between metabolic products and substrates added (acetate, total lactate and Fe^0^). Error bars show duplicates absolute deviation from average.

The electron balance shows that part of the lactate oligomers were used for fermentation to carboxylates (Figure 3). Lactate monomers and acetate in the substrate comprised about 70% of the total electrons added (total lactate and acetate). Substrate conversion was 75 ± 4% in the control experiment suggesting slight hydrolysis in the absence of nZVI. However, substrate conversion was 87–88% under conditions of n-caproate production where hydrolysis of lactate oligomers was estimated to be > 2-times higher than without nZVI (Supplementary Table 2). When hydrogen was initially added (experiment III), substrate conversion was similar to without nZVI (≤81%) (Supplementary Figure 3 and Supplementary Table 3).

The microbiome produced n-butyrate efficiently with an electron selectivity of 91 ± 1% in the control experiment. At 1 g nZVI⋅L^–1^, n-caproate electron selectivity peaked to 28 ± 1% while n-butyrate selectivity was reduced to 57 ± 1%. Propionate reached a maximum electron selectivity of 32 ± 7% at 5 g nZVI⋅L^–1^. Carbon selectivity for n-butyrate reached 54 ± 6% in the control experiment and 15 ± 1% for n-caproate at 1 g nZVI⋅L^–1^ while n-valerate was produced at much lower proportions (3 ± 1% at 3.5 g nZVI⋅L^–1^). Carbon was diverged toward propionate at low selectivities (2–4% at 0–2 g nZVI⋅L^–1^) although it raised up to 10 ± 1% and 21 ± 6% when adding 3.5 and 5 g nZVI⋅L^–1^, respectively. Carbon selectivity for CO_2_ ranged between 37 and 52% by the end of the chain elongation phase (days 0–7) and showed a decreasing trend during the hydrogen consumption phase (Supplementary Figure 2). Evident decrease in CO_2_ carbon selectivity (8–15% lower) was observed in incubations displaying n-valerate and n-caproate formation.

### Experiment IV—Lactate Enantiomers Conversion to n-Butyrate at Different Rates Boosted by nZVI

**FIGURE 4:**
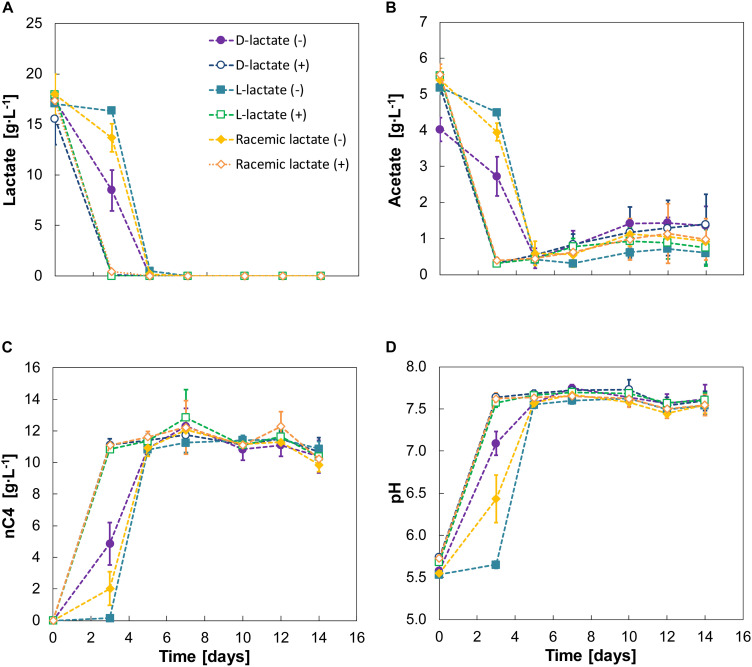
Experiment IV—Substrate **(A,B)**, n-butyrate **(C)** and pH **(D)** profiles for D-lactate, L-lactate and racemic lactate conversion. A positive or negative sign between braces indicate absence (–) or presence (+) of nZVI (added at 1 g⋅L^–1^). Error bars indicate ± one standard deviation.

To evaluate the effect of the two different lactate enantiomers on microbial chain elongation, separate experiments (experiment IV) with either D-lactate, L-lactate or a racemic mixture were carried out with (1 g⋅L^–1^) and without nZVI. Both enantiomers were converted into n-butyrate (Figure 4) at high electron selectivities (96–100%) (Supplementary Figure 6). The efficient conversion of lactate into n-butyrate probably resulted in a shortage of electron donor for further elongation to n-caproate. Addition of nZVI did not affect the product spectrum of fermentation but resulted in lactate depletion within 3 days irrespectively of the enantiomer supplied. No odd-chain carboxylates (propionate, n-valerate, n-heptanoate) were produced.

Although lactate was converted to n-butyrate in all cases, different lag phases were evident for the two enantiomers. D-lactate was converted earlier than L-lactate with the latter being barely consumed during the first 3 days. Racemic lactate showed a reduced the lag phase compared to fermentation of L-lactate alone. During this first 3 days, D-lactate and the racemic mixture showed conversion rates of 2.13 ± 0.8 and 1.2 ± 1.1 g⋅L^–1^⋅d^–1^, respectively. After the lag phase, however, L-lactate conversion was observed at 8.59 ± 0.1 g⋅L^–1^⋅d^–1^ and was almost depleted within 2 days (days 3–5). Conversion rates on days 3–5 were 4.6 ± 1.2 g⋅L^–1^⋅d^–1^ for D-lactate and 7.1 ± 0.7 g⋅L^–1^⋅d^–1^ for racemic lactate. n-butyrate production rates for days 0–3 were 0.67 ± 0.35 and 1.62 ± 0.45 for the racemic mixture and D-lactate, respectively. From days 3 to 5, n-butyrate productivities from L-lactate, D-lactate and the racemic mixture were, respectively, 5.34 ± 0.15, 3.0 ± 0.56 and 4.46 ± 0.49 g⋅L^–1^⋅d^–1^. The enantiomeric excess for L-lactate was still high (88 ± 7%) on day 3 and D-lactate was measured to be 1.07 ± 0.64 g⋅L^–1^ (6 ± 3% of total lactate). In general, enantiopure lactate (either D- or L-lactate) was racemized during fermentation while racemic equilibrium was maintained when feeding racemic lactate (Supplementary Figure 7). Chain elongation was followed by a hydrogen consumption phase with concomitant CO_2_ consumption and acetate formation (Supplementary Figure 8 and Figure 4B). H_2_ and CO_2_ consumption resulted in decreased CO_2_ carbon selectivity from 34 ± 1% by the end of the chain elongation phase to 29 ± 2% by the end of the experiment.

### nZVI Shapes Microbiome Composition

**FIGURE 5:**
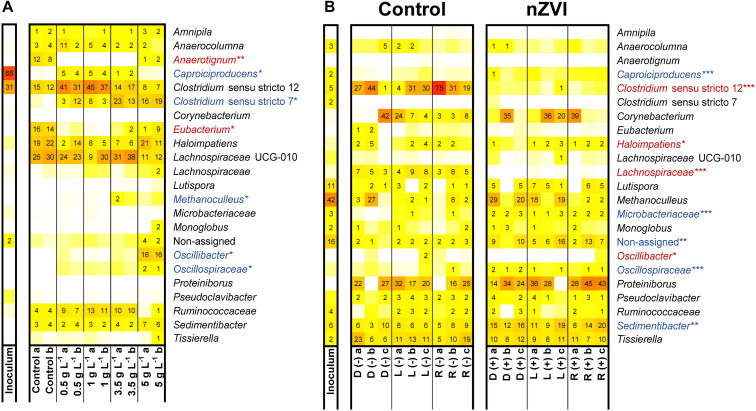
Microbiome composition in experiment II **(A)** and experiment IV **(B)**. Experiment IV used D-lactate (D), L-lactate (L) or racemic lactate (R) in the absence (–) or presence (+) of nZVI (1 g⋅L^–1^) (**B**). Blue and red shaded taxa indicate whether relative abundance was found positively or negatively correlated with nZVI presence (*** indicates *p* < 0.0005; ** indicates *p* < 0.001; * indicates *p* < 0.01).

Composition of microbiomes from experiment II was affected by nZVI presence and concentration (Supplementary Figure 9A). nZVI presence was negatively related to the occurrence of *Anaerotignum* (only member *Anaerotignum propionicum* sp. ASV23; 100% identity, formerly *Clostridium propionicum*) and *Eubacterium* species (Figure 5A). Several taxa were enriched at different nZVI concentrations. *Clostridium sensu stricto* 12 species were enriched (32–47% relative abundance) in n-caproate producing conditions compared to incubations without nZVI (12–16%). This genus was predominantly represented (65–86% of counts) by *Clostridium tyrobutyricum* sequences (ASV1) which showed relative abundances of 21–32% (samples 0.5–1 g nZVI⋅L^–1^). *Clostridium luticellarii* (ASV29) from the same genus was also enriched to relative abundances 3–7% when n-caproate was produced while it remained <1% in the other conditions. Three sequences were classified as *Caproiciproducens* species of which *Caproiciproducens* sp. ASV37 was identified only in n-caproate producing incubations (3–4% relative abundance) and showed low similarity to *C. galactitolivorans* BS-1 (90% identity). *Caproiciproducens* sp. ASV37 (*p* < 0.01), *Clostridium tyrobutyricum* sp. ASV1 (*p* < 0.001) and *Clostridium luticellarii* sp. ASV29 (*p* < 0.001) were positively correlated with n-caproate concentrations. The genus *Ruminococcaceae* was correlated with both n-valerate (*p* < 0.01) and n-caproate (*p* < 0.001). At ≥ 3.5 g nZVI⋅L^–1^, relative abundance for *Clostridium sensu stricto* 12 was reduced (8–18% relative abundance) while *Clostridium novyi* sp. ASV7, only member of the genus *Clostridium sensu stricto* 7, was enriched to relative abundances of 14–24% (Figure 5A). At 5 g nZVI⋅L^–1^, *Oscillibacter* sp. ASV22 (98.5% identity *Oscillibacter valericigenes*) and *Sedimentibacter* sp. ASV10 (97% identity *Sedimentibacter saalensis*) reached 17 and 7% relative abundance, respectively. *Clostridium sensu stricto* 7, *Oscillibacter*, *Oscillospiraceae* and *Sedimentibacter* species were among the highly abundant genera related with high nZVI and propionate production.

In experiment IV, feeding enantiopure or racemic sodium lactate did not influence the microbial community composition whereas nZVI presence and acetate formation during the hydrogen consumption did (Supplementary Figure 9B). Species of *Clostridium sensu stricto* 12, *Haloimpatiens*, unclassified *Lachnospiraceae* and *Oscillibacter* were negatively related with nZVI (Figure 5B). Notably, *Clostridium sensu stricto* 12 species (being > 77% *C. tyrobutyricum* sp. ASV1) showed decreased relative abundances from up to 75 to <1% with nZVI addition. On the other hand, *Sedimentibacter* species along with the less abundant genus *Caproiciproducens*, unclassified *Microbacteraceae* and unclassified *Oscillospiraceae* showed a positive correlation with nZVI. Other highly abundant genera such as *Proteiniborus* and *Corynebacterium*, with *Corynebacterium provencense* sp. ASV4 as the only member, showed no significant correlation with nZVI (Figure 5B). Identified genera correlating with acetate formation were *Eubacterium* (*p* < 0.001), *Lachnospiraceae* UCG-010 (*p* < 0.001), *Lutispora* (*p* < 0.01) and *Methanoculleus* (*p* < 0.0005). *Corynebacterium provencense* was negatively correlated with acetate formation (*p* < 0.0005). *Methanoculleus* sequences (*Methanoculleus palmolei* sp. ASV6; 100% identity) were highly abundant in the inoculum sample and were also detected in the incubations. However, methane was not detected in our experiments and *M. palmolei* is reported to not grow on lactate (Zellner et al., 1998). Since the reactor used as inoculum source was unattended for some time when the inoculum aliquot for experiment IV was taken, it is possible that leftover sequences from inactive *M. palmolei* were amplified from the incubations.

## Discussion

### Alkaline Hydrolysis of Lactate Esters With nZVI May Improve Chain Elongation

Dosing nZVI resulted in higher substrate availability due to chemical hydrolysis of lactate oligomers which translated into higher substrate conversion to carboxylates. Here we report that hydrolysis of lactate esters can occur purely chemically due to the high reactivity of nZVI. During iron corrosion, pH close to metallic surfaces can be up to 2 units higher than the bulk pH depending on reaction rates and mixing conditions (Kahyarian et al., 2017). This ΔpH is expected to be higher in highly reactive ZVI nanoparticles (Eqs. 10 and 11), likely promoting alkaline de-esterification of lactate oligomers (Eq. 12). At alkaline conditions, OH^–^ ions cleave the ester bonds of lactate oligomers unzipping them to yield lactate as the final hydrolytic product (Ivanova et al., 2000). This observed effect of nZVI could be used to promote hydrolysis of PLA-based bioplastics (Ivanova et al., 2000). Chemical hydrolysis was probably limited by nZVI reacting only partially and by being used for side reactions other than oligomers hydrolysis e.g., hydrogen formation (Eq. 10). Assuming that OH^–^ ions from anaerobic corrosion of nZVI (Eq. 11) react with lactyl lactate (2 mol lactyl lactate^–^⋅ Fe^0^ ^–1^) to produce lactate (Eq. 12), 1 g nZVI⋅L^–1^ could hydrolyze 50% of the lactate oligomers. Instead, hydrolysis was observed at about 30%. nZVI was added in excess at 5 g nZVI⋅L^–1^ with incomplete hydrolysis obtained despite nZVI corrosion was still feasible at the near-neutral pH observed (Eq. 11 at pH 7; ΔG′ = −10.6 kJ⋅reaction^–1^). Hydrolysis of lactate oligomers was enhanced with nZVI under fermentation conditions probably by a combination of chemical and biological hydrolysis (Li et al., 2017) as well as continuous lactate consumption. Since microbial chain elongation is shown to be dependent on lactate availability (Kucek et al., 2016), continuous, gradual hydrolysis of lactate oligomers could have enhanced chain elongation.

### nZVI Affects Fermentation Conditions Shaping Microbiome Composition and Lactate Metabolism

Addition of nZVI created specific fermentation conditions that influenced microbiomes composition, lactate conversion rates and final product spectrum. In experiment II, the presence of nZVI was negatively correlated with n-butyrate concentrations (*p* < 0.01) while n-caproate showed a positive correlation (*p* < 0.01) (Supplementary Figure 10). Moreover, dose-dependent effects were observed on substrate conversion rates. Initial lactate conversion occurred at higher rates with nZVI doses of 0.5–2 g⋅L^–1^ compared to ≥ 3.5 g⋅L^–1^ (days 0–2). Overall, lactate was depleted within 7–9 days. Under anaerobic conditions, nZVI corrosion occurs rapidly in the first ∼24 h to gradually level out reaching almost full depletion in 2–7 days depending on the system conditions (Fan et al., 2016; Qin et al., 2018). The associated decrease in ORP (Wang et al., 2020) and increased pH due to the rapid initial nZVI corrosion probably shortened the lag phase compared to fermentation without nZVI. Adding 0.5–2 g nZVI⋅L^–1^ improved substrate conversion and chain elongation to n-valerate and n-caproate. Although elongation of acetate and n-butyrate is thermodynamically more favorable with nZVI as the electron donor (Eqs. 15 and 16) compared to lactate (Eqs. 4–8) or hydrogen (Eqs. 17 and 18), lactate was available at higher amounts with nZVI being a secondary electron source. Considering that bacteria may derive electrons from the conversion of lactate to acetate (4 e^–^mol⋅lactate^–1^; Eq. 2) to drive the RBO pathway, Fe^0^ in added nZVI (2 e^–^mol⋅Fe^0–1^; Eqs. 10 and 11) could have contributed to 1–10% (0.5–5 g nZVI⋅L^–1^) of electrons in total lactate. Both n-valerate and n-caproate production correlated (p < 0.01) with increased substrate conversion (Supplementary Figure 10) indicating that chain elongation was related to increased use of lactate as electron donor. Hydrolyzed lactate seemed to be rapidly used for chain elongation since chain elongation products showed sustained production over time despite lactate monomers were depleted (Figure 2). Several bacteria harboring genes involved in lactate oxidation (lactate dehydrogenases, Ldh) and the RBO pathway such as *C. tyrobutyricum* (Wu et al., 2017), *C. luticellarii* (Poehlein et al., 2018) and members of the family *Ruminococcaceae* (Tao et al., 2017) including *Caproiciproducens* species (e.g., Bengelsdorf et al., 2019) were enriched in these conditions. *C. tyrobutyricum* is known as an efficient n-butyrate producer (Wu et al., 2017) but is not reported to produce longer carboxylates. The capability of *C. luticellarii* (Petrognani et al., 2020), *Ruminococcaceae* (Yang et al., 2020), and *Caproiciproducens* (Bengelsdorf et al., 2019) organisms to produce carboxylates with more than 4 carbons and their high correlation with n-valerate and n-caproate formation suggest their involvement in chain elongation.

**FIGURE 6:**
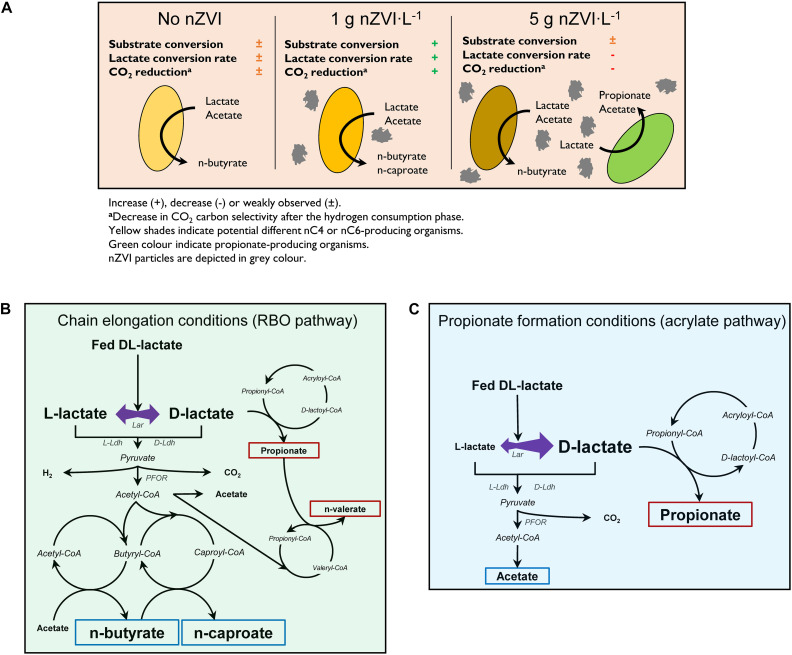
Proposed effects of nZVI on lactate-based chain elongation **(A)**, enantiomeric proportions during lactate conversion to elongated carboxylates **(B)** and to propionate through the acrylate pathway **(C)**. Blue and red boxes depict even- and odd-chained carboxylates, respectively. Abbreviations: Lar, lactate racemase; D-Ldh, D-lactate dehydrogenase; L-Ldh, L-lactate dehydrogenase; PFOR, pyruvate:ferredoxin oxidoreductase. **(B,C)** Adapted from Schweiger and Buckel (1984); Kuchta and Abeles (1985), Hino and Kuroda (1993), and Angenent et al. (2016).

At high nZVI concentrations (5 g⋅L^–1^), other potential n-butyrate producers such as *Oscillibacter*, unclassified *Oscillospiraceae* and *Sedimentibacter* species were enriched. These taxa have also been enriched in bioelectrochemical systems (Breitenstein et al., 2002; Lin et al., 2018; Dessì et al., 2021) and *Oscillibacter* species in ethanol-based chain elongation with nZVI (Fu et al., 2020; Wang et al., 2020). However, nZVI caused higher pH conditions and directed the conversion of residual lactate toward propionate formation. Known propionic acid bacteria (PAB) grow optimally at pH values close to neutrality (Hettinga and Reinbold, 1972; Seeliger et al., 2002). *Clostridium novyi* was markedly enriched in this conditions, an organism reported to use the acrylate pathway for propionate formation (Reichardt et al., 2014). *C. novyi* strains also possess key genes for H_2_/CO_2_ uptake (Ryan et al., 2008) which might have facilitated its growth in the presence of nZVI over *Anaerotignum propionicum*, a well-known PAB using the acrylate pathway (Akedo et al., 1983; Kuchta and Abeles, 1985). Propionate concentrations correlated with increasing nZVI concentrations (*p* < 0.001) (Supplementary Figure 10). Here, n-valerate formation was not promoted despite the high propionate concentrations. Hydrogen partial pressure (P_*H*__2_) did not seem to influence lactate chain elongation or propionate formation since similar P_*H*__2_ values and behavior were observed with and without nZVI addition in experiment II. Other reports show improved lactate conversion to MCC with hydrogen addition (Nzeteu et al., 2018; Wu et al., 2019). This was not the case in our study even when P_*H*__2_ up to 2.6 atm (experiment II) were observed. H_2_ addition in experiment III did not alter product spectrum from lactate and H_2_ did not elongate acetate or n-butyrate in the absence of lactate. Although not tested in this study, acetate and n-butyrate elongation with nZVI (Eqs. 15 and 16) may be more feasible than with H_2_ as the electron donor (Eqs. 17 and 18). Figure 6A summarizes the observed effects of nZVI on the overall conversion process.

Isomerization of L-lactate to D-lactate has been suggested to be a required rate-limiting step prior chain elongation (Kucek et al., 2016). Therefore, an enrichment of D-lactate induced with nZVI addition (Li et al., 2017) would be expected to improve chain elongation rates. This was probably based on the fact that *M. elsdenii* is reported to produce even-chains from D-lactate and propionate/odd-chains from L-lactate (Hino and Kuroda, 1993). In contrast, D-lactate is the substrate for the acrylate pathway in PAB (Akedo et al., 1983; Schweiger and Buckel, 1984). Despite the enantiomer fed, however, racemase activity in *M. elsdenii* resulted in similar carboxylates proportions (Hino and Kuroda, 1993). This is in line with our observations that both D-lactate and L-lactate are racemized in chain-elongating microbiomes regardless of nZVI addition (Figure 6B). Thus, improved chain elongation rates in the presence of nZVI are not related with an excess of D-lactate. Instead, D-lactate excess was associated to the dominance of propionate formation most likely through the acrylate pathway (Figure 6C).

nZVI has been shown to decrease ORP creating favorable conditions for ethanol-based chain elongation (Wang et al., 2020). The same authors showed that nZVI increase conductivity which was suggested to facilitate direct electron transfer and MCC formation. In the present work, n-valerate and n-caproate production may have been partly promoted by nZVI donating electrons to bacteria. Electron uptake may support a high intracellular Fd_red_ pool facilitating anaerobic lactate oxidation. Anaerobic lactate oxidation is an energetically unfavored reaction that requires bacteria to use confurcating Ldh [Ldh and electron transferring flavoprotein (EtfAB) complex] to drive the endergonic reduction of NAD (E°′_NAD/NADH_ = −320 mV; Thauer et al., 1977) with lactate (E°′_pyruvate/lactate_ = −190 mV) by simultaneously oxidizing Fd_red_ (Weghoff et al., 2015; Buckel and Thauer, 2018). Under anaerobic conditions, nZVI (E°′Fe^2+^/Fe^0^ = −469 mV; Rickard and Iii, 2007) could transfer electrons directly to ferredoxins (E°′Fd_ox_/Fd_red_ = −500 −−398 mV; Thauer et al., 1977; Li and Elliott, 2016) when ferredoxins are present extracellularly (Marshall et al., 2017). Alternatively, ferredoxin can be reduced through membrane-bound or cytoplasmic reversible electron-bifurcating systems (Buckel and Thauer, 2018) *via* direct or H_2_-mediated electron transfer. For instance, direct electron transfer may involve the membrane-bound Rnf complex using electrochemically-driven Na^+^/H^+^ gradients and NADH for Fd_ox_ reduction (Gong et al., 2020). H_2_-mediated ferredoxin reduction could occur through the cytoplasmic Hyd complex oxidizing H_2_ to provide Fd_red_ and NADH for microbial metabolism (Buckel and Thauer, 2013). Reverse electron transport by the membrane-bound Ech complex uses H_2_ and Na^+^/H^+^ gradients to form Fd_red_ (Hedderich and Forzi, 2005) being therefore influenced by both direct and H_2_-mediated electron transfer. Genes encoding for Rnf and Ech are reported to be present in several chain-elongating bacteria with Rnf being more widespread than Ech (Liu et al., 2020). As mentioned earlier, nZVI could have contributed to 1–10% (0.5–5 g nZVI⋅L^–1^) of electrons in total lactate. However, when considering the confurcating lactate oxidation to pyruvate step (lactate + 2 Fd_red_ + 2 NAD → pyruvate + 2 Fd_ox_ + 2 NADH; 2 e^–^mol⋅lactate^–1^ and 2 e^–^mol⋅Fd_red_^–1^), nZVI contributions through Fd_red_ would be doubled. Therefore, small amounts of nZVI could significantly influence lactate-to-pyruvate flux and the formation of elongated products. nZVI could also influence energy conservation and butyryl-CoA dehydrogenase in the RBO pathway (Angenent et al., 2016). Extracellular Fe^2+^ reduction may also be coupled to lactate oxidation in a more energetically favorable reaction (Eq. 3).

The addition of nZVI to fermentation of both enantiopure and racemic lactate substrates resulted in faster lactate conversion to n-butyrate. Notably, the microbiome composition was drastically changed by nZVI addition with *Clostridium* (*C. tyrobutyricum*) and unclassified *Lachnospiraceae* being outcompeted by other potential n-butyrate producing bacteria. *Sedimentibacter* isolates are reported to use pyruvate and amino acids, but not lactate, with production of SCC, mainly acetate and n-butyrate without H_2_ formation (Zhang et al., 1994; Breitenstein et al., 2002; Imachi et al., 2016). Some strains, however, possess putative proteins involved in lactate metabolism (section “Lactate Metabolism and Racemization”). *Sedimentibacter* species have been suggested to be electroactive as they were enriched in bioelectrochemical systems (Vilajeliu-Pons et al., 2016) and graphene-amended anaerobic digestion (Lin et al., 2018). Additionally, some species are involved in extracellular electron transfer for Fe^3+^ reduction (Burkhardt et al., 2011). Therefore, *Sedimentibacter* capability to interact with external electron donors/sinks and iron availability might have been advantageous to thrive in the presence of nZVI. This may be the case also for other taxa enriched with nZVI addition. Other highly abundant species from the genera *Corynebacterium* and *Proteiniborus* are able to metabolize lactate to n-butyrate but showed low or no correlation with nZVI addition. *Corynebacterium provencense* genome harbors genes that encode a predicted lactate utilization protein with a ferredoxin-type domain (NCBI Reference Sequence WP_110482617.1). Genes involved in the RBO pathway are also present in *C. provencense* and other *Corynebacterium* species (Takeno et al., 2013; Kittl et al., 2018). A *Proteiniborus* isolate from anaerobic digestion was predicted to harbor lactate dehydrogenases and produce acetate (including Wood-Ljungdahl pathway), propionate (acrylate pathway) and n-butyrate (butyrate kinase pathway) (Maus et al., 2017). Differences in inoculum composition and/or Na^+^ levels between experiment IV using sodium lactate (∼4.8 g Na^+^⋅L^–1^) and experiment II using concentrated lactic acid (∼0.13 g Na^+^⋅L^–1^) (section “Experiment II and II—nZVI Addition to a Mixture of Lactate Monomers and Oligomers Steers to Microbial N-Caproate Formation”) may have contributed to relatively different microbiomes developed in the two experiments.

Although nZVI may inactivate gram-negative more severely than gram-positive bacteria (Diao and Yao, 2009), taxa enriched with nZVI addition include species showing both positive and negative gram stains. Therefore, selective inhibition based on cell wall composition was not clearly observed. Instead, the enriched taxa may have thrived probably due to changed pH, ORP and iron availability conditions and by being involved in extracellular electron transfer as discussed in this section.

### Improved Carbon Recovery Through Chain Elongation and Carbon Dioxide Recapture

Carbon dioxide could be reduce to acetate with electrons from nZVI (Eq. 13) or H_2_ (Eq. 14). Hydrogen consumption was observed in the last days of incubation in experiments II (days 7–12) and IV (days 3–12 and days 5–12 with and without nZVI, respectively). However, different substrate conversions observed in experiment II affected apparent H_2_ and CO_2_ formation/consumption during this phase. No significant correlation was found between H_2_ consumption and CO_2_ or acetate formation during the hydrogen consumption phase but acetate formed during this phase was positively related with substrate conversion, n-valerate and n-caproate concentrations (all with *p* < 0.01) (Supplementary Figure 10). This suggests that part of this acetate was formed as a side product of chain elongation. In days 7–12, CO_2_ was produced at similar amounts in all conditions. Assuming that lactate was the electron donor used for elongation to nC5/nC6, higher amounts of CO_2_ due to lactate decarboxylation are expected when chain elongation activity was observed (0.5–2 g nZVI⋅L^–1^). This indicates that CO_2_ may have been recovered with nZVI or H_2_. Decreases in CO_2_ carbon selectivity during the hydrogen consumption phase were related with higher substrate conversions (*p* < 0.001) (toward chain elongation) and less CO_2_ release (*p* < 0.01) (Supplementary Figure 10). CO_2_ could be incorporated into acetate, n-valerate and/or n-caproate thereby improving carbon recovery in the chain elongation process. Increased relative abundance in chain-elongating conditions (0.5–2 g nZVI⋅L^–1^) was observed for *C. luticellarii* which may use the Wood-Ljungdahl pathway to take up electrons, H_2_ and/or CO_2_ coupled to chain elongation (Poehlein et al., 2018; Petrognani et al., 2020). *Caproiciproducens* and *Ruminococcaceae* species were also enriched under these same conditions but their capability to utilize CO_2_/H_2_/electrons for chain elongation remains to be studied. In contrast, homoacetogenic activity was more clearly observed in experiment IV where acetate formation correlated with H_2_ (*p* < 0.01) and CO_2_ (*p* < 0.05) consumption during the hydrogen consumption phase (Supplementary Figure 11). Of the CO_2_ formed from lactate conversion, 24 ± 5% in absence and 20 ± 10% in presence of nZVI was consumed. Hence, nZVI presence showed no significant correlation with H_2_/CO_2_ consumption nor with acetate production (Supplementary Figure 11). Overall, several genera comprising homoacetogenesis-related genes such as *Clostridium*, *Eubacterium*, *Lachnospiraceae*, *Proteiniborus*, and *Corynebacterium* (Singh et al., 2019) were identified in the incubations. Further studies using omics or pure cultures could be done to clarify the role of aforementioned organisms in H_2_/CO_2_ utilization and chain elongation.

Soluble iron concentrations by the end of the experiments were below 3 mg⋅L^–1^ (<0.5% of iron added) in all the incubations with nZVI. Thus, we could not quantify the extent of nZVI reaction based on soluble iron. The formation of insoluble iron species may account for such low soluble iron concentrations.

### Lactate Metabolism and Racemization

Even though lactate-to-acetate ratios tested here could in principle yield n-caproate (Table 1, Eq. 8), n-butyrate was the main product with stoichiometry shown in Eq. 4 [average of L(-), D(-) and R(-) log-phase]. Comparable n-butyrate yields were observed in experiment II in the absence of nZVI. A similar reaction stoichiometry was reported for *Clostridium acetobutylicum* converting lactate and acetate to n-butyrate (Diez-Gonzalez et al., 1995). This experimental reaction stoichiometry was slightly more thermodynamically feasible during the incubations compared to the proposed reaction in equation 5 but was still less favorable compared to n-caproate formation (Supplementary Figure 12). n-butyrate over n-caproate formation may be a common outcome of batch lactate chain elongation with reactor microbiomes. Although n-caproate formation is more energetically favorable than n-butyrate (Table 1), it requires one extra RBO cycle with the concomitant higher ATP yield (Angenent et al., 2016). Based on the microbial ecology theory on so called r- and K-selection, conditions of excess substrate (high food-to-microorganism ratio) select for r-strategists displaying high-flux metabolism (q_S_^max^, μ^max^) while K-strategists exhibiting high-yield metabolism (Y_X,ATP_, K_S_) thrive under substrate-limiting conditions (Andrews and Harris, 1986). Following this principle, fast-growing n-butyrate producers may dominate in batch microbiomes while more efficient n-caproate producers would thrive under substrate limitation. This principle is evidenced here by the late n-caproate production in our experiments (day 5 onward) and C. *tyrobutyricum* outcompeting *Caproiciproducens*-related species that were highly abundant in the inoculum derived from a continuous chain elongation reactor (section “nZVI Shapes Microbiome Composition”). Presence of n-butyrate favors n-caproate producers in (repeated) batch reactors fermenting food waste (Nzeteu et al., 2018; Contreras-Dávila et al., 2020) which is also in line with the aforementioned theory (Andrews and Harris, 1986). Despite that the pH-independent propionate production reaction (Eq. 9) was thermodynamically favored during the incubations (Supplementary Figure 12), growth of PAB was constrained by the initial acidic pH conditions applied (Hettinga and Reinbold, 1972; Stinson and Naftulin, 1991) and high propionate concentrations observed at 5 g nZVI⋅L^–1^ were related to high pH and residual lactate (section “nZVI Affects Fermentation Conditions Shaping Microbiome Composition and Lactate Metabolism”).

Lactate oxidation is catalyzed by stereospecific Ldh. Prompt utilization of D-lactate is usually observed since D-Ldh is a constitutive enzyme in many microorganisms while L-Ldh is inducible by the presence of L-lactate (Kato et al., 2010). When only one of the two stereospecific Ldh is present, utilization of both enantiomers may still proceed by lactate isomerization through lactate racemase (Lar). In the acrylate pathway, D-lactate is selectively converted through lactoyl-CoA and acryloyl-CoA to propionate (Akedo et al., 1983; Schweiger and Buckel, 1984; Kuchta and Abeles, 1985). This requirement of D-lactate may explain the isomerization of L-lactate to a large D-lactate excess when propionate was formed, most likely through the acrylate pathway present in *C. novyi*. In rumen fermentation, where the acrylate pathway is dominant, an excess of D-lactate was also associated with higher propionate formation (Counotte and Prins, 1981). The role of lactate racemization in other lactate-consuming bacteria is not quite clear. Lactate racemization was first observed in butyrate-producing bacteria in early reports (Tatum et al., 1936; Christensen et al., 1939). Lar was determined to be an excretable enzyme acting on the dissociated lactate form with optimal pH 5 (Christensen et al., 1939; Dennis and Kaplan, 1965). Although lactate isomerization itself does not give bacteria any energetic advantage (ΔG° = −0.4 kJ⋅mol^–1^; Eq. 1), a racemic mixture of lactate might result in higher chain-elongating rates in the case that both enantiomers are metabolized simultaneously. However, we observed higher conversion rates with enantiopure substrates [D-lactate (days 0–3) and L-lactate (days 3–5), section “Experiment IV—Lactate Enantiomers Conversion to n-Butyrate at Different Rates Boosted by nZVI”). Whether D- or L-lactate was the required substrate for chain elongation is not clear from our experiments due to the observed lactate racemization. The n-caproate producer *Rumminococcaceae* bacterium CPB6 possess both L-Ldh and D-Ldh and the expression of L-ldh in recombinant *E. coli* was shown to enhance lactate conversion by 17-fold (Yang et al., 2020). However, properties of the D-Ldh were not studied. High L-lactate conversion rates observed in the present study could be related to lower pyruvate inhibition of L-Ldh compared to D-Ldh similarly to PAB *P. freudenreichii* (Crow, 1986). Alternatively, a higher concentration of Lar induced at high L/D lactate ratios (Goffin et al., 2005) may increase lactate racemization and, consequently, chain elongation rates. Genes encoding Lar are present in several bacteria including chain-elongating organisms such as *M. elsdenii* and *E. limosum* (Desguin et al., 2014). A Pfam query (El-Gebali et al., 2019) for Lar (N-terminal domain, PF09861.10) showed that most of the genera detected in this study include organisms with putative Lar such as *Caproiciproducens*, *Clostridium* (including *C. tyrobutyricum*, *C. luticellarii*), *Corynebacterium*, *Eubacterium*, *Haloimpatiens*, *Lachnoclostridium*, *Lachnospiraceae*, *Oscillibacter*, *Rumminococcaceae* (including *Rumminococcaceae* bacterium CPB6) and *Sedimentibacter*. Other genera include the PAB *Propionibacterium* and *Veillonella* (Supplementary Material). An hypothetical role of Lar in energy conservation *via* transmembrane proton gradient formation has been suggested (Desguin et al., 2017). Pure culture studies using lactate-elongating organisms and mutants lacking Lar genes could be useful to elucidate the relevance of lactate racemization and enantiomers metabolism under different operational conditions.

### Outlook

Reaction rates and MCC selectivity observed here could be improved by testing nZVI in pH-controlled experiments. Mildly acidic conditions not only favor both iron corrosion and chain elongation but may decrease the likelihood of iron precipitates formation (Kahyarian et al., 2017) and allow complete nZVI utilization. Spent nZVI precipitates could be collected and recycled to Fe^0^ and, when FeCO_3_ is present, to reduced one-carbon compounds (CO, CH_4_) through reductive calcination (Lux et al., 2019). Soluble Fe^2+^ can also be electrochemically recovered as goethite (FeOOH) (Nguyen and Ahn, 2018) and may be recycled together with the other precipitates. Carbon monoxide (CO) could be looped back into the chain elongation process as additional electron and carbon source. Although nZVI may be a costly additive, its use in chain elongation may be feasible when dealing with complex substrates. nZVI addition to waste activated sludge supplied with ethanol increased substrate conversion and n-caproate selectivity (Wang et al., 2020). Importantly, some of the effects triggered with nZVI can be replicated with (bio)electrochemistry. Production of OH^–^ ions to hydrolyze lactate oligomers or PLA bioplastics could be achieved by applying cathodic current, avoiding the need of Fe^0^ or external caustic input. Biological chain elongation is also susceptible to external control under so-called electro-fermentation conditions (Moscoviz et al., 2016) where relatively small proportions of electrons (compared to electrons in fermentable substrate) supplied or subtracted through electrodes affect microbial metabolism. Although fermentable substrates (lactate and acetate) were the main source of electrons in our experiments, small contributions to this electron pool coming from nZVI (0.3–3% total substrate; 1–20% electrons transferred) could have influenced specific oxidation-reduction reactions significantly e.g., Fd_red_-dependent lactate oxidation, pyruvate decarboxylation and energy conservation (section “nZVI Affects Fermentation Conditions Shaping Microbiome Composition and Lactate Metabolism”). Therefore, cathodic electrons could be continuously supplied under controlled pH conditions to steer propionate formation and chain elongation of even or odd carboxylates.

## Conclusion

Lactate-based chain elongation microbiomes, lactate conversion rates and product spectrum were affected at different nZVI doses. The effects of nZVI on chain elongation were related to hydrolysis of lactate oligomers, changes in pH and possibly by acting as additional electron donor. The metabolism arising under specific conditions (e.g., pH, lactate and nZVI concentrations) determined both lactate enantiomeric proportions and product spectrum. Lactate was racemized in chain-elongating microbiomes while D-lactate excess was observed during propionate production through the acrylate pathway. Our results suggest that feeding D-lactate to continuous reactors would not necessarily translate into higher chain elongation rates due to lactate racemization. Carbon recovery into carboxylates was increased after hydrogen consumption which was presumably coupled with carbon dioxide recapture into acetate and elongated carboxylates. Fermentation conditions imposed by the presence and concentration of nZVI could be replicated with bioelectrochemical systems to control carboxylates production under continuous operation.

## Data Availability Statement

The datasets presented in this study can be found in online repositories. The names of the repository/repositories and accession number(s) can be found in the article/Supplementary Material.

## Author Contributions

CC-D designed the study, carried out the data analysis, and drafted the manuscript. JE carried out the experiments and supported the data analysis. CB supported in the review and editing of the manuscript. DS conceptualized, designed and supervised the study, and supported in the review and editing of the manuscript. All authors read and approved the final manuscript.

## Conflict of Interest

The authors declare that the research was conducted in the absence of any commercial or financial relationships that could be construed as a potential conflict of interest.

## Footnotes

1 https://doi.org/10.4121/14236922

2 https://www.ebi.ac.uk/ena
