# Supplementary material for: nZVI Impacts Substrate Conversion and Microbiome Composition in Chain Elongation From D- and L-Lactate Substrates
Source: Front Bioeng Biotechnol. 2021 Jun 15;9:666582. doi: 10.3389/fbioe.2021.666582 (PMC8239352; doi:10.3389/fbioe.2021.666582)
Supplement: Supplementary file 2 [file Data_Sheet_2.docx]

*Supplementary material*

nZVI impacts substrate conversion and microbiome composition in lactate-based chain elongation from D- and L-lactate substrates

Carlos A. Contreras-Dávila^1^, Johan Esveld^1^, Cees J.N. Buisman^1^ and David P.B.T.B. Strik^1,^*

^1^ Environmental Technology, Wageningen University & Research, Bornse Weilanden 9, 6708 WG, Wageningen, the Netherlands.

***** Correspondence: david.strik@wur.nl

3 tables; 12 figures.

**Analytical methods**

Lactate (both D- and L-lactate monomers together), succinate and formate were measured by HPLC (Contreras-Dávila et al., 2020). Lactate enantiomers (D-lactate and L-lactate) were separated and quantified with a chiral column Astec CLC-L (15 cm × 4.6 mm, 5 µm) (Supelco) using isocratic HPLC (Thermo Scientific Dionex UltiMate 3000 RS, Thermo Fischer) with UV-detection at 254 nm (Dionex UltiMate VWD-3400). The mobile phase was 5 mM cupric sulfate at 1 mL/min and injection volume of 30 µL. Temperature in the column oven was 25°C. Chromatography data were analyzed with Chromeleon software (v6.8).

Fatty acids and alcohols, i.e. straight-chain fatty acids (C2-C8), isobutyrate, isovalerate (both 2- and 3-methylbutanoic acids together), isocaproate (4- methyl-pentanoic acid) and straight-chain alcohols (C1-C6) were quantified by gas chromatography (Agilent 7890B) using FID-detection at 240°C and a HP-FFAP column (25m x 0.32mm x 0.50µm). The carrier gas was helium at 1.25 mL∙min^−1^ for the first 3 min and 2 mL∙min^−1^ for the rest of the run. Injection volume was 1 µL (split injection 1:25) and injection valve temperature kept constant at 250°C. Oven temperature ramp was: 60°C for the first 3 min; 21°C∙min^-1^ up to 140°C; 8°C∙min^-1^ up to 150°C and constant for 1.5 min; 120°C∙min^-1^ up to 200°C and constant for 1.25 min; 120°C∙min^-1^ up to 240°C and constant for 3 minutes.

Nitrogen, oxygen, methane and carbon dioxide were measured using GC (Shimadzu GC-2010, Japan) equipped with TCD detector and parallel column setup (gas split 1:1) of Agilent PoraBOND Q (50 m x 0.53 mm x 10 µm) and Molsieve 5A (25 m x 0.53 mm x 50 µm). Carrier gas was helium at 22.5 mL∙min^−1^. The oven temperature was 80 °C and TCD 150 °C. Hydrogen was measured with an HP 5890 gas chromatograph by injecting 100 μl of gas-sample on a Molsieve 5A column (30 m × 0.53 mm × 25 μm) with thermal conductivity detection (TCD). The oven temperature was 40 °C and µ-TCD 150 °C. The carrier gas was argon with a flow rate of 20 mL∙min^−1^.

**Calculations**

Substrate conversion was calculated based on electron equivalents (e^-^ eq) in final products divided by substrate added (total lactate + acetate) (eq. S1): propionate (14 e^-^ eq/mol), n-butyrate (20 e^-^ eq/mol), n-valerate (26 e^-^ eq/mol), n-caproate (32 e^-^ eq/mol) and hydrogen (2 e^-^ eq/mol) related to lactate (12 e- eq/mol) and acetate (8 e^-^ eq/mol). Electron selectivity for product *i* is related to the total products measured (eq. S2). Carbon selectivity is regarded as the fraction of carbon in product *i* relative to carboxylates and total carbon dioxide produced (eq. S3). Total carbon dioxide (CO_2_) includes gaseous CO_2_ and aqueous inorganic carbon (IC_aq_) species (H_2_CO_3_, HCO_3_^-^ and CO_3_^2-^) according to eq. S4 where γ_CO2_ = CO_2_ headspace fraction; P_tot_ = total pressure in batch bottle; and V_liq_ = liquid volume. IC_aq_ was calculated after de Leeuw et al. (2020) (De Leeuw et al., 2020) (eq. S5 and S6) where: [H^+^] = 10^-pH^; K_a1_ = 10^-6.35^; K_a2_ = 10^-10.33^; and K_H_ = 29.41 atm/M^2^. Lastly, the proportions of L- or D-lactate were expressed as enantiomeric excess with respect to L-lactate according to eq. S7. Enantiomeric excess is a measurement of purity used for chiral substances where enantiopure solutions have an excess of 100%. An enantiomeric excess of 90% for L-lactate denotes that the other 10% is a racemic mixture (1:1) of both enantiomers. To calculate D-lactate enantiomeric excess, the difference between D-lactate and L-lactate concentrations replaces the numerator in eq. S7. Enantiomeric excess is expressed with respect to L-lactate unless stated otherwise.

$Substrate conversion=100\times\frac{e^{-}{eq}_{products}}{e^{-}{eq}_{substrate}}$ (S1)

$Electron selectivity=100\times\frac{e^{-}{eq}_{i}}{e^{-}{eq}_{products}}$ (S2)

$Carbon selectivity=100 \times\frac{{mol C}_{i}}{{mol C}_{products}}$ (S3)

$Total {CO}_{2}= \frac{\gamma_{{CO}_{2}} \left( - \right) \times P_{tot} \left( atm \right) \times V_{gas}}{V_{liq} \times R \times T}+ {IC}_{aq}$ (S4)

${IC}_{aq}= \frac{\left[ H_{2}{CO}_{3} \right] \times({{[H}^{+}]}^{2} + {K_{a}}_{1} \times\left[ H^{+} \right] + {K_{a}}_{2} \times{K_{a}}_{1})}{{[H^{+}]}^{2}}$ (S5)

$[H_{2}{CO}_{3}]= \frac{\gamma_{{CO}_{2}} \left( - \right) \times P_{tot} (atm)}{K_{H}}$ (S6)

$Enantiomeric excess=100 \times\frac{(L\text{-}lactate-D\text{-}lactate)}{(L\text{-}lactate+D\text{-}lactate)}$ (S7)

Table S1. Gibbs energy of formation values used for thermodynamics calculations.

| **Compound** | **G⁰*f* (kJ∙mol^-1^)** | **S_298.1_ (cal∙mol^-1^∙K^-1^)** | **Reference** |
| --- | --- | --- | --- |
| L-lactate |  | 34.3 | (Huffman et al., 1940) |
| D-lactate |  | 34 |  |
| lactate | -517.1 |  | (Kleerebezem and Van Loosdrecht, 2010) |
| acetate | -369.4 |  |  |
| propionate | -361.1 |  |  |
| n-butyrate | -352.7 |  |  |
| n-valerate | -344.3 |  |  |
| n-caproate | -336 |  |  |
| carbon dioxide | -394.4 |  |  |
| lactyl lactic acid | -727.8 |  | (De Clercq et al., 2018) |
| water | -237.2 |  | (Kleerebezem and Van Loosdrecht, 2010) |
| OH^-^ | -237.2 |  | G⁰*f_OH-_ =* G⁰*f_H2O_* from water equilibrium |
| Fe^2+^ | -90.53 |  | (Rickard and Iii, 2007) |

Table S2. Experiment II - Substrate conversion and oligomers hydrolysis at different nZVI concentrations.

Table S3. Substrate conversion and lactate oligomers hydrolysis in experiment III.

Figure S1. Experiment II – Hydrogen (A) and carbon dioxide (B) gasses partial pressures; total carbon dioxide (C) and carbon selectivity (D) in lactate-based chain elongation incubations with different nZVI doses. Total carbon dioxide as inorganic carbon (IC) refers to ICaq + CO_2_ gas (normalized for liquid volume) as in eq. S4-S5 (C). Carbon selectivity by the end of the chain elongation phase (days 0 to 7) and by the end of the experiment (days 0 to 12) (D). Error bars show duplicates absolute deviation from the average.

Figure S2. Experiment II - n-valerate (nC5) concentration profiles at different nZVI doses. Error bars show duplicates absolute deviation from the average.

Figure S3. Experiment III - Lactate-based chain elongation without (control) and with additional hydrogen (0.45 and 1.2 atm). Hydrogen (0.75 atm) was also tested as sole electron donor with acetate and n-butyrate as electron acceptors with no chain elongation activity observed. CO_2_ was added at 0.3 atm in all cases and incubations lasted for 15 days. Error bars show duplicates absolute deviation from the average.

Figure S4. Experiment II - Enantiomeric excess during lactate conversion at different nZVI doses showing that L-lactate was racemized close to equimolar concentrations (excess 0%) during chain elongation at 0-2 g nZVI∙L^-1^. In contrast, D-lactate was in excess on days 5-7 coinciding with an increased propionate formation at 5 g nZVI∙L^-1^. Negative values indicate an excess of the D-lactate enantiomer. Error bars show duplicates absolute deviation from the average.

Figure S5. Experiment II - Calculated (A) and measured (B) acetate concentrations increase during the hydrogen consumption phase. Calculated acetate formation was based on hydrogen consumption assuming stoichiometry 4 H_2_ + 2 CO_2_ → acetate. Error bars show duplicates absolute deviation from the average.

Figure S6. Experiment IV - Net conversion of lactate enantiomers and acetate by the end of the experiments with enantiopure or racemic lactate. D-lactate (D), L-lactate (L) or racemic lactate (R) with (+) and without (-) nZVI (1 g∙L^-1^). Error bars indicate ±one standard deviation.

Figure S7. Experiment IV - Enantiomeric excess during lactate enantiomers conversion to n-butyrate. Enantiomeric excess was calculated with respect to D-lactate for the D- and racemic lactate experiments. The figure shows that L-lactate was to a limited extent isomerized to D-lactate during the 3-days lag phase. Enantiopure lactate was racemized to equilibrium during conversion. Lactate concentration were below the quantification limit (10 mg∙L^-1^) on day 5 but enantiomeric excess was close to the racemic equilibrium based on the chromatograms areas. Error bars indicate ±one standard deviation.

Figure S8. Experiment IV - Hydrogen (A) and carbon dioxide (B) partial pressures; total inorganic carbon (C) and carbon selectivity (D) in lactate-based chain elongation incubations fed enantiopure or racemic lactate. D-lactate (D), L-lactate (L) or racemic lactate (R) in the absence (-) or presence (+) of nZVI (1 g∙L-1). Total carbon dioxide as inorganic (IC) refers to ICaq + CO_2_ gas (normalized for liquid volume) as in eq. 4-5 (D). Carbon selectivity by the end of the chain elongation phase (day 0 to day 5 and day 3 without and with nZVI, respectively) and by the end of the experiment (days 0 to 14). Error bars show ±one standard deviation.

Figure S9. Distance-based redundancy analysis (dbRDA) for microbiomes from experiment II (A) and experiment IV (B). The analyses used Bray-Curtis dissimilarity index; ASVs relative abundance as response variables; and environmental parameters as explanatory variables. Environmental parameters considered were: nZVI presence, nZVI concentration, substrate conversion and final concentrations of propionate, nC4, nC5 and nC6 for (A); nZVI presence, initial D- and L-lactate concentrations, final nC4 concentrations and acetate production during the hydrogen consumption phase for (B). Concentration ellipses depict confidence intervals with α = 0.05. Significance code: ‘***’ associated with a variable at P <0.0005; ‘**’ associated with a variable at P <0.001; and ‘*’ associated with a variable at P <0.01.


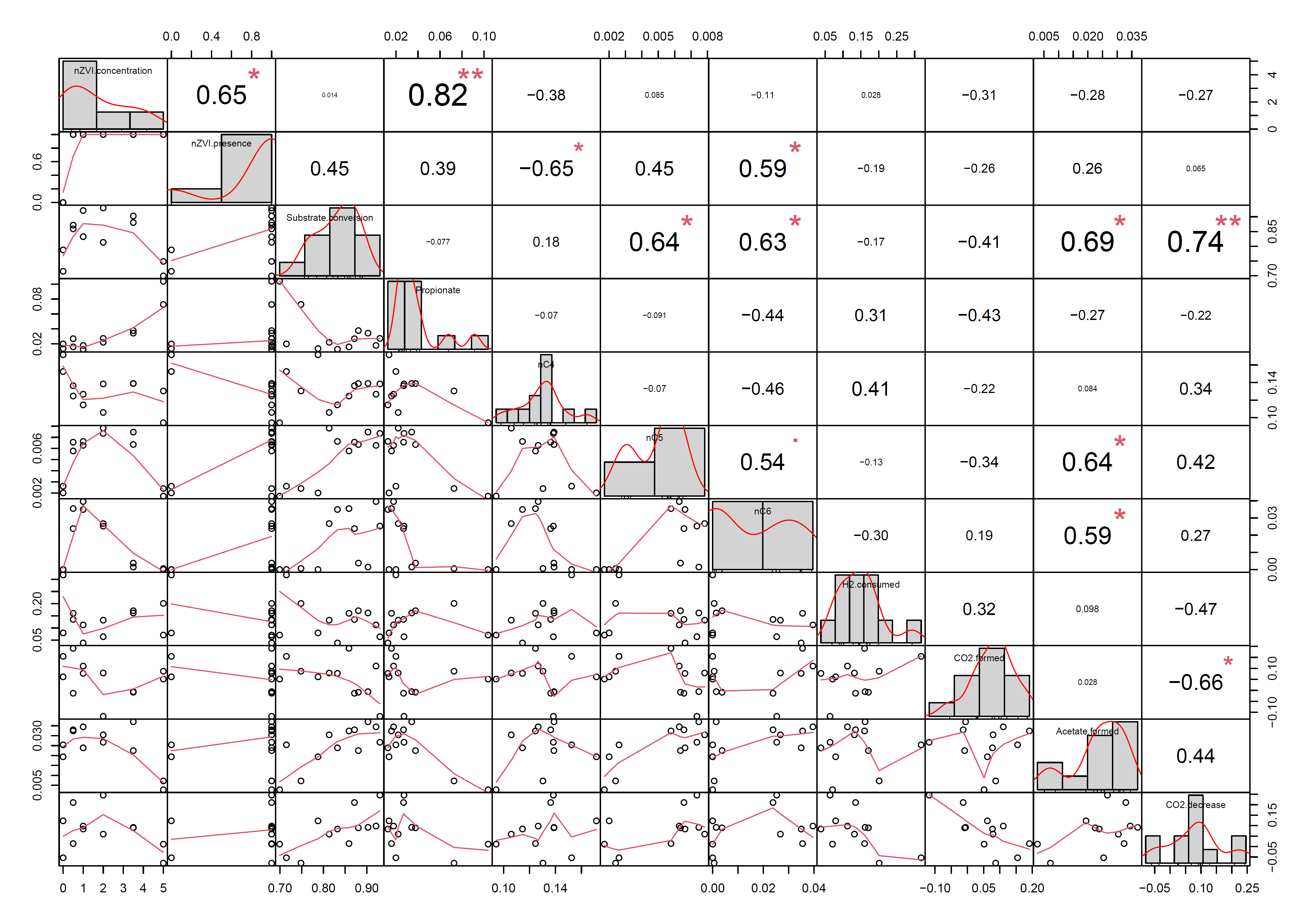


Figure S10. Experiment II – Correlation matrix for physicochemical parameters based on Spearman’s rank correlation. The distribution of each variable is shown on the diagonal; the bivariate scatter plots with a fitted line below the diagonal; and Spearman’s correlation coefficients above the diagonal. Correlations significance level is depicted with stars (‘***’ indicates p-values <0.0005; ‘**’ indicates p-values <0.001; ‘*’ indicates p-values <0.01 and ‘▪’ indicates p-values <0.05). Molar concentrations for all metabolites was used. H_2_ consumed, CO_2_ and acetate formed refer to their change in molar concentrations during the hydrogen consumption phase (days 7 to 12). CO_2_ decrease refers to the decrease in CO_2_ carbon selectivity during the hydrogen consumption phase (1 – CO_2_-selectivity_day12_/CO_2_-selectivity_day7_).


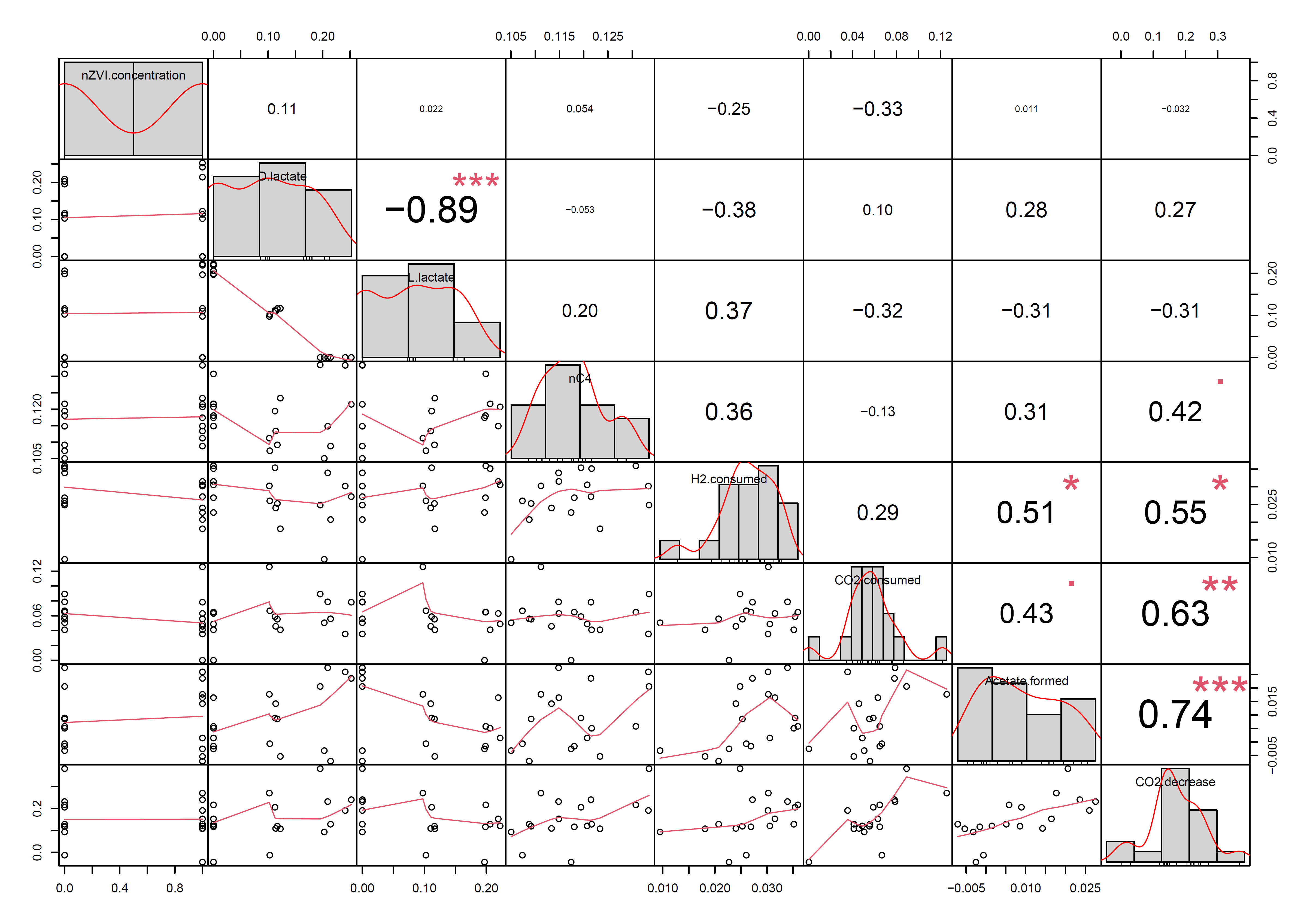


Figure S11. Experiment IV – Correlation matrix for physicochemical parameters based on Spearman’s rank correlation. The distribution of each variable is shown on the diagonal; the bivariate scatter plots with a fitted line below the diagonal; and Spearman’s correlation coefficients above the diagonal. Correlations significance level is depicted with stars (‘***’ indicates p-values <0.0005; ‘**’ indicates p-values <0.001; ‘*’ indicates p-values <0.01 and ‘▪’ indicates p-values <0.05). Molar concentrations were used for all metabolites. H_2_ and CO_2_ consumed as well as acetate formed refer to their change in concentrations during the hydrogen consumption phase (days 3 to 14 and days 5 to 14 with and without nZVI, respectively). CO_2_ decrease refers to the decrease in CO_2_ carbon selectivity during the hydrogen consumption phase (1 – CO_2_-selectivity_day12_/CO_2_-selectivity_day7_).

Figure S12. Actual Gibbs free energy of reaction (∆G^'^_r_) adjusted for variations in metabolites and proton concentrations over time for experiments with 0 (A), 1 (B) and 5 (B) g nZVI∙L^-1^ (section 3.2). Water concentrations was assumed 55 M. Reactions stoichiometry are described in Table 1 equations 4, 5, 8 and 9 for n-butyrate, n-butyrate-Exp, n-caproate and Propionate-acetate, respectively. Gibbs free energy values were calculated according to ∆G^'^_r_ = ∆G°_r_ + R∙T∙lnQ using values given in Table S1.

**REFERENCES**

Contreras-Dávila, C. A., Carrión, V. J., Vonk, V. R., Buisman, C. N. J., and Strik, D. P. B. T. B. (2020). Consecutive lactate formation and chain elongation to reduce exogenous chemicals input in repeated-batch food waste fermentation. *Water Res.* 169. doi:10.1016/j.watres.2019.115215.

De Clercq, R., Dusselier, M., Makshina, E., and Sels, B. F. (2018). Catalytic Gas-Phase Production of Lactide from Renewable Alkyl Lactates. *Angew. Chemie - Int. Ed.* 57, 3074–3078. doi:10.1002/anie.201711446.

De Leeuw, K. D., De Smit, S. M., Van Oossanen, S., Moerland, M. J., Buisman, C. J. N., and Strik, D. P. B. T. B. (2020). Methanol-Based Chain Elongation with Acetate to n-Butyrate and Isobutyrate at Varying Selectivities Dependent on pH. *ACS Sustain. Chem. Eng.* 8, 8184–8194. doi:10.1021/acssuschemeng.0c00907.

Huffman, H. M., Ellis, E. L., and Borsook, H. (1940). Thermal Data XI. The Heat Capacities and Entropies of Guanidine Carbonate, Glutamic Acid Hydrochloride, Ornithine Dihydrochloride, D-Lactic Acid and L-Lactic Acid. *J. Am. Chem. Soc.* 62, 297–299. doi:10.1021/ja01859a016.

Kleerebezem, R., and Van Loosdrecht, M. C. M. (2010). A generalized method for thermodynamic state analysis of environmental systems. *Crit. Rev. Environ. Sci. Technol.* 40, 1–54. doi:10.1080/10643380802000974.

Rickard, D., and Iii, G. W. L. (2007). Chemistry of Iron Sulfides. *Chem. Rev.* 107, 514–562. doi:10.1021/cr0503658.
